# Crystal structure of *N*′-[(*E*)-(4-chloro­phen­yl)(phen­yl)methyl­idene]-4-methyl­benzene­sulfono­hydrazide

**DOI:** 10.1107/S2056989014026723

**Published:** 2015-01-01

**Authors:** J. Balaji, S. Prabu, J. J. F. Xavier, P. Srinivasan

**Affiliations:** a Department of Physics, UCEP, Panruti 607 106, TamilNadu, India; b Department of Chemistry, UCEP, Panruti 607 106, TamilNadu, India

**Keywords:** crystal structure, benzene­sulfono­hydrazide, hydrogen bonding, condensation reaction, centrosymmetric dimers

## Abstract

The title compound, C_20_H_17_ClN_2_O_2_S, was obtained by a condensation reaction between 4-chloro­benzo­phenone and tosyl hydrazide. The plane of the methyl-substituted benzene ring forms dihedral angles of 20.12 (12) and 78.43 (13)° with those of the chlorine-substituted benzene ring and the benzene ring, respectively, with the last two rings forming a dihedral angle of 67.81 (13)°. The chlorine substituent was also found to be 0.868 (2):0.132 (2) disordered over these two rings. In the crystal, mol­ecules are linked through pairs of N—H⋯O hydrogen bonds, giving centrosymmetric cyclic dimers [graph set *R*_2_^2^(8)], which are linked by weak C—H⋯O and C—H⋯Cl inter­actions into a chain structure which extends along the *a*-axis direction.

## Related literature   

Benzo­phenone and its derivatives have been investigated extensively for their biological activities such as anti-fungal and anti-inflammatory, see: Khanum *et al.* (2004 ▸). For similar structures, see: Ajani *et al.* (2010 ▸); Gerdemann *et al.* (2002 ▸); Kutzke *et al.* (2000 ▸); Shen *et al.* (2012 ▸); Zhang (2011 ▸).
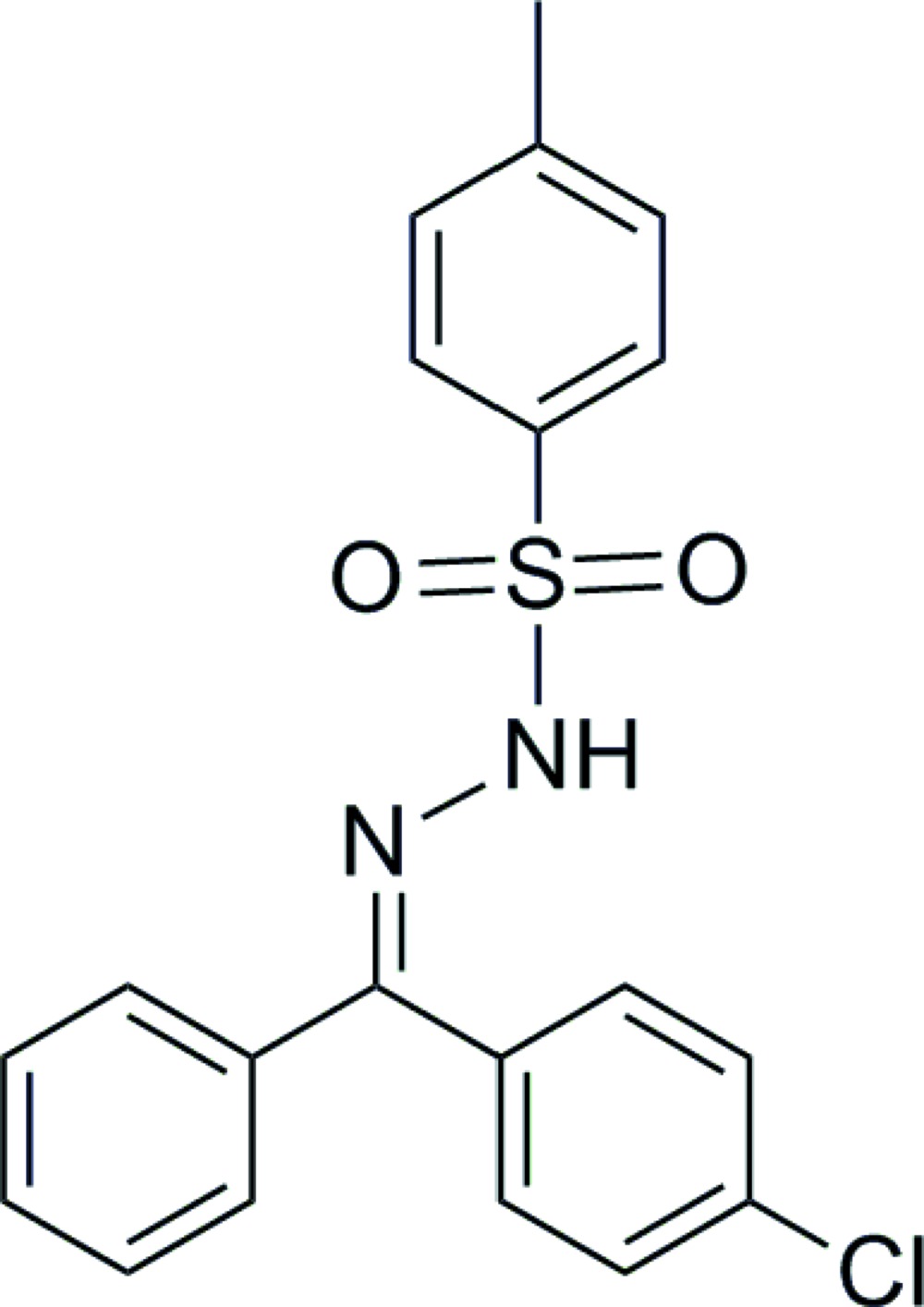


## Experimental   

### Crystal data  

C_20_H_17_ClN_2_O_2_S
*M* *_r_* = 384.87
Monoclinic, 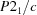

*a* = 12.6808 (6) Å
*b* = 9.3857 (5) Å
*c* = 16.3974 (7) Å
β = 106.187 (2)°
*V* = 1874.22 (16) Å^3^
*Z* = 4
Mo *K*α radiation
μ = 0.33 mm^−1^
*T* = 293 K
0.35 × 0.30 × 0.25 mm

### Data collection  

Bruker Kappa APEXII CCD diffractometer
Absorption correction: multi-scan (*SADABS*; Sheldrick, 1996 ▸) *T*_min_ = 0.891, *T*_max_ = 0.930
21401 measured reflections
3300 independent reflections
2416 reflections with *I* > 2σ(*I*)
*R*_int_ = 0.032

### Refinement  

*R*[*F* ^2^ > 2σ(*F* ^2^)] = 0.039
*wR*(*F* ^2^) = 0.105
*S* = 1.06
3300 reflections
253 parameters
4 restraints
H atoms treated by a mixture of independent and constrained refinement
Δρ_max_ = 0.35 e Å^−3^
Δρ_min_ = −0.33 e Å^−3^

### 

Data collection: *APEX2* (Bruker, 2004 ▸); cell refinement: *APEX2* and *SAINT* (Bruker, 2004 ▸); data reduction: *SAINT* and *XPREP* (Bruker, 2004 ▸); program(s) used to solve structure: *SIR92* (Altomare *et al.*, 1993 ▸); program(s) used to refine structure: *SHELXL97* (Sheldrick, 2008 ▸); molecular graphics: *ORTEP-3 for Windows* (Farrugia, 2012 ▸) and *Mercury* (Macrae *et al.*, 2008 ▸); software used to prepare material for publication: *SHELXL97*.

## Supplementary Material

Crystal structure: contains datablock(s) I. DOI: 10.1107/S2056989014026723/zs2319sup1.cif

Structure factors: contains datablock(s) I. DOI: 10.1107/S2056989014026723/zs2319Isup2.hkl

Supporting information file. DOI: 10.1107/S2056989014026723/zs2319Isup3.cml

**Fig. 1.**
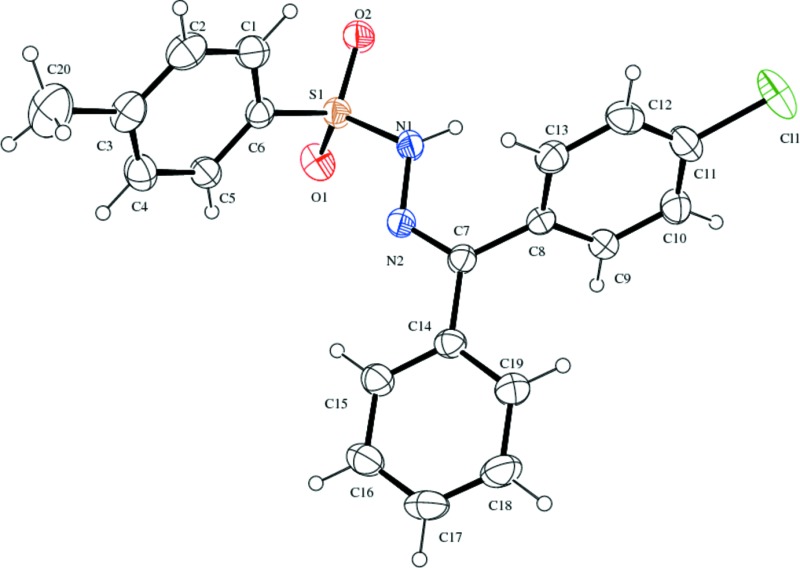
The molecular structure of the title compound showing the atom labelling scheme. The displacement ellipsoids are drawn at the 30% probability level

. DOI: 10.1107/S2056989014026723/zs2319fig1.tif
The mol­ecular structure of the title compound showing the atom labelling scheme. The displacement ellipsoids are drawn at the 30% probability level

**Fig. 2.**
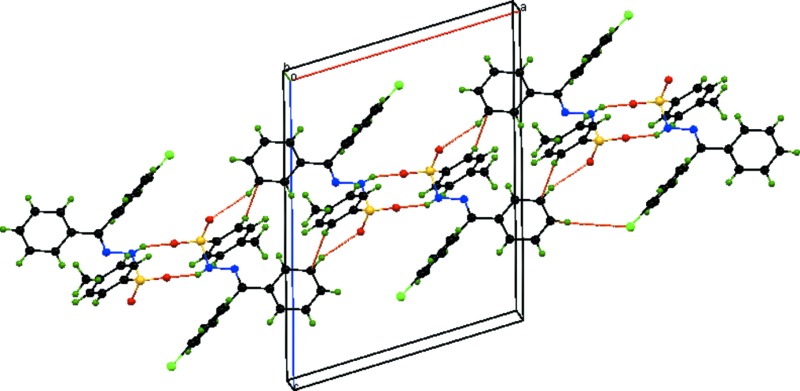
A view of the crystal packing of the title compound. The various hydrogen bonds are indicated by dashed lines (see Table 1 for details).

. DOI: 10.1107/S2056989014026723/zs2319fig2.tif
A view of the crystal packing of the title compound. The various hydrogen bonds are indicated by dashed lines (see Table 1 for details).

CCDC reference: 1037752

Additional supporting information:  crystallographic information; 3D view; checkCIF report

## Figures and Tables

**Table 1:** Hydrogen-bond geometry (, )

*D*H*A*	*D*H	H*A*	*D* *A*	*D*H*A*
N1H1*A*O2^i^	0.88(2)	2.19(2)	3.024(3)	160(2)
C10H10Cl1^ii^	0.93	2.76	3.476(7)	134
C16H16O1^iii^	0.93	2.54	3.339(3)	145

Symmetry codes: (i) 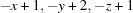; (ii) 
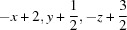
; (iii) 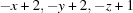.

## supplementary crystallographic information

### S1. Chemical context

Currently the hydrazones have attracted considerable attention due to their biological activities and a number of crystal structures of these compounds have been reported (Ajani *et al.*, 2010); Gerdemann *et al.*, 2002; Kutzke *et al.*, 2000); Zhang, 2011; Shen *et al.*, 2012). Benzo­phenone and its derivatives have also been extensively investigated for their biological activities such as anti-fungal and anti-inflammatory (Khanum *et al.*, 2004). In view of the importance of these analogs, the title compound, C_20_H_17_ClN_2_O_2_S, was synthesized in a Schiff base condensation reaction between 4-chloro­benzo­phenone and tosyl hydrazide and its structure is reported herein.

In this compound (Fig. 1) the benzene ring (C1–C6) forms dihedral angles of 20.12 (12) and 78.43 (13)° with the chlorine-substituted benzene ring (C8–C13) and the benzene ring (C14–C19), respectively. The molecule is twisted, with the dihedral angle between the two benzene rings (C8–C13 and C14–C19) of the parent moiety being 67.81 (13)°. In the crystal, molecules are linked through inter­molecular N1—H···O2^i^ hydrogen-bond pairs (Table 1) giving centrosymmetric cyclic dimers [graph set *R*^2^_2_(8)] which are linked by weak C—H···O and C—H···Cl inter­actions into a chain structure which extends along *a* (Fig. 2).

### S2. Synthesis and crystallization

4-Chloro­benzo­phenone (0.15g, 1 mmol) and tosyl hydrazide (0.186g, 1 mmol) were dissolved in ethanol (50 ml). The reaction mixture was heated under reflux for 3 hr and cooled gradually to room temperature. Crystals suitable for X-ray diffraction analysis were obtained by slow room temperature evaporation of the solution containing the compound.

### S3. Refinement

All H atoms were positioned geometrically and treated as riding on their parent atoms with C—H = 0.93 Å (aromatic) or 0.96 Å (methyl) and N1—H = 0.89±2 Å with *U*_iso_(H) = 1.2 *U*_eq_(N, C_aromatic_) or 1.5 *U*_eq_(C_methyl_). The chlorine substituent was also found to be disordered over the C8–C13 (Cl1) and C14–C19 (Cl1') rings of the original benzo­phenone moiety [occupancy factors 0.868 (2):0.132 (2), respectively].

### Crystal data

**Table:**

C_20_H_17_ClN_2_O_2_S	*F*(000) = 800
*M**_r_* = 384.87	*D*_x_ = 1.364 Mg m^−^^3^
Monoclinic, *P*2_1_/*c*	Mo *K*α radiation, λ = 0.71073 Å
Hall symbol: -P 2ybc	Cell parameters from 6873 reflections
*a* = 12.6808 (6) Å	θ = 2.5–25.2°
*b* = 9.3857 (5) Å	µ = 0.33 mm^−^^1^
*c* = 16.3974 (7) Å	*T* = 293 K
β = 106.187 (2)°	Block, brown
*V* = 1874.22 (16) Å^3^	0.35 × 0.30 × 0.25 mm
*Z* = 4	

### Data collection

**Table:**

Bruker Kappa APEXII CCD diffractometer	3300 independent reflections
Radiation source: fine-focus sealed tube	2416 reflections with *I* > 2σ(*I*)
Graphite monochromator	*R*_int_ = 0.032
ω and φ scans	θ_max_ = 25.0°, θ_min_ = 2.5°
Absorption correction: multi-scan (*SADABS*; Sheldrick, 1996)	*h* = −15→15
*T*_min_ = 0.891, *T*_max_ = 0.930	*k* = −11→11
21401 measured reflections	*l* = −18→19

### Refinement

**Table:**

Refinement on *F*^2^	Secondary atom site location: difference Fourier map
Least-squares matrix: full	Hydrogen site location: inferred from neighbouring sites
*R*[*F*^2^ > 2σ(*F*^2^)] = 0.039	H atoms treated by a mixture of independent and constrained refinement
*wR*(*F*^2^) = 0.105	*w* = 1/[σ^2^(*F*_o_^2^) + (0.0351*P*)^2^ + 1.1726*P*] where *P* = (*F*_o_^2^ + 2*F*_c_^2^)/3
*S* = 1.06	(Δ/σ)_max_ = 0.001
3300 reflections	Δρ_max_ = 0.35 e Å^−^^3^
253 parameters	Δρ_min_ = −0.33 e Å^−^^3^
4 restraints	Extinction correction: *SHELXL97* (Sheldrick, 2008), Fc^*^=kFc[1+0.001xFc^2^λ^3^/sin(2θ)]^-1/4^
Primary atom site location: structure-invariant direct methods	Extinction coefficient: 0.0025 (7)

### Special details

**Table:**

Geometry. All e.s.d.'s (except the e.s.d. in the dihedral angle between two l.s. planes) are estimated using the full covariance matrix. The cell e.s.d.'s are taken into account individually in the estimation of e.s.d.'s in distances, angles and torsion angles; correlations between e.s.d.'s in cell parameters are only used when they are defined by crystal symmetry. An approximate (isotropic) treatment of cell e.s.d.'s is used for estimating e.s.d.'s involving l.s. planes.
Refinement. Refinement of *F*^2^ against ALL reflections. The weighted *R*-factor *wR* and goodness of fit *S* are based on *F*^2^, conventional *R*-factors *R* are based on *F*, with *F* set to zero for negative *F*^2^. The threshold expression of *F*^2^ > σ(*F*^2^) is used only for calculating *R*-factors(gt) *etc*. and is not relevant to the choice of reflections for refinement. *R*-factors based on *F*^2^ are statistically about twice as large as those based on *F*, and *R*- factors based on ALL data will be even larger.

### Fractional atomic coordinates and isotropic or equivalent isotropic displacement parameters (Å^2^)

**Table:**

	*x*	*y*	*z*	*U*_iso_*/*U*_eq_	Occ. (<1)
C1	0.6473 (2)	0.5896 (3)	0.50383 (16)	0.0567 (7)	
H1	0.5830	0.6014	0.5198	0.068*	
C2	0.6980 (3)	0.4588 (3)	0.51169 (18)	0.0650 (8)	
H2	0.6676	0.3825	0.5334	0.078*	
C3	0.7928 (3)	0.4383 (3)	0.48812 (18)	0.0632 (7)	
C4	0.8369 (2)	0.5531 (3)	0.45737 (17)	0.0612 (7)	
H4	0.9013	0.5410	0.4416	0.073*	
C5	0.7885 (2)	0.6855 (3)	0.44933 (16)	0.0510 (6)	
H5	0.8200	0.7621	0.4288	0.061*	
C6	0.69263 (19)	0.7028 (3)	0.47211 (14)	0.0445 (6)	
C7	0.81124 (18)	1.0259 (2)	0.65813 (15)	0.0430 (6)	
C8	0.73683 (18)	1.0797 (3)	0.70683 (14)	0.0429 (6)	
C9	0.7223 (2)	1.2238 (3)	0.71776 (16)	0.0511 (6)	
H9	0.7612	1.2898	0.6953	0.061*	
C10	0.6505 (2)	1.2706 (3)	0.76179 (17)	0.0581 (7)	
H10	0.6405	1.3675	0.7691	0.070*	
C11	0.5943 (2)	1.1708 (3)	0.79449 (16)	0.0587 (7)	
C12	0.6081 (2)	1.0282 (3)	0.78491 (17)	0.0605 (7)	
H12	0.5692	0.9624	0.8077	0.073*	
C13	0.6794 (2)	0.9831 (3)	0.74156 (15)	0.0525 (6)	
H13	0.6895	0.8859	0.7353	0.063*	
C14	0.93096 (18)	1.0480 (3)	0.68959 (15)	0.0453 (6)	
C15	0.9995 (2)	1.0041 (3)	0.64206 (17)	0.0541 (7)	
H15	0.9696	0.9608	0.5897	0.065*	
C16	1.1112 (2)	1.0234 (3)	0.6711 (2)	0.0632 (8)	
H16	1.1566	0.9927	0.6388	0.076*	
C17	1.1549 (2)	1.0880 (3)	0.7477 (2)	0.0699 (9)	
H17	1.2283 (17)	1.100 (4)	0.769 (2)	0.084*	
C18	1.0896 (2)	1.1329 (3)	0.7961 (2)	0.0723 (8)	
H18	1.1203	1.1770	0.8481	0.087*	
C19	0.9777 (2)	1.1124 (3)	0.76732 (17)	0.0610 (7)	
H19	0.9331	1.1421	0.8005	0.073*	
C20	0.8462 (3)	0.2933 (4)	0.4951 (3)	0.1014 (12)	
H20A	0.8929	0.2890	0.4580	0.152*	
H20B	0.7905	0.2213	0.4791	0.152*	
H20C	0.8893	0.2776	0.5526	0.152*	
N1	0.66524 (16)	0.9315 (2)	0.55792 (13)	0.0548 (6)	
N2	0.77696 (15)	0.9565 (2)	0.58850 (13)	0.0498 (5)	
O1	0.67110 (15)	0.9520 (2)	0.40485 (12)	0.0639 (5)	
O2	0.51226 (13)	0.8526 (2)	0.44334 (11)	0.0599 (5)	
S1	0.62845 (5)	0.86959 (7)	0.46061 (4)	0.0477 (2)	
Cl1	0.50538 (8)	1.22428 (12)	0.85037 (6)	0.0880 (4)	0.868 (2)
Cl1'	1.2814 (4)	1.1131 (6)	0.7865 (4)	0.0616 (19)	0.132 (2)
H1A	0.6174 (19)	0.988 (2)	0.5711 (16)	0.061 (8)*	

### Atomic displacement parameters (Å^2^)

**Table:**

	*U*^11^	*U*^22^	*U*^33^	*U*^12^	*U*^13^	*U*^23^
C1	0.0582 (16)	0.0650 (18)	0.0486 (15)	−0.0090 (14)	0.0175 (12)	−0.0045 (13)
C2	0.078 (2)	0.0537 (18)	0.0595 (17)	−0.0106 (15)	0.0136 (15)	0.0068 (14)
C3	0.073 (2)	0.0506 (17)	0.0568 (17)	0.0071 (14)	0.0038 (14)	0.0010 (14)
C4	0.0578 (17)	0.0611 (18)	0.0649 (18)	0.0115 (14)	0.0173 (14)	−0.0015 (14)
C5	0.0493 (15)	0.0498 (15)	0.0558 (16)	0.0009 (12)	0.0179 (12)	0.0001 (12)
C6	0.0467 (14)	0.0476 (14)	0.0371 (13)	−0.0008 (11)	0.0081 (11)	−0.0058 (11)
C7	0.0414 (13)	0.0402 (13)	0.0454 (14)	0.0014 (10)	0.0087 (11)	−0.0039 (11)
C8	0.0381 (12)	0.0476 (14)	0.0408 (13)	−0.0034 (10)	0.0072 (10)	−0.0069 (11)
C9	0.0473 (14)	0.0517 (15)	0.0577 (16)	−0.0038 (12)	0.0200 (12)	−0.0072 (12)
C10	0.0584 (16)	0.0554 (16)	0.0617 (17)	0.0062 (13)	0.0191 (14)	−0.0084 (14)
C11	0.0449 (15)	0.085 (2)	0.0483 (15)	0.0102 (14)	0.0167 (12)	0.0013 (15)
C12	0.0563 (16)	0.075 (2)	0.0517 (16)	−0.0085 (15)	0.0174 (13)	0.0071 (14)
C13	0.0557 (15)	0.0526 (15)	0.0478 (15)	−0.0043 (12)	0.0122 (12)	−0.0031 (12)
C14	0.0399 (13)	0.0422 (13)	0.0510 (15)	0.0005 (10)	0.0081 (11)	0.0014 (11)
C15	0.0471 (14)	0.0533 (16)	0.0627 (17)	0.0036 (12)	0.0165 (13)	0.0001 (13)
C16	0.0457 (15)	0.0585 (17)	0.088 (2)	0.0035 (13)	0.0225 (15)	0.0049 (16)
C17	0.0399 (15)	0.0563 (18)	0.104 (3)	−0.0040 (14)	0.0041 (17)	0.0068 (17)
C18	0.0513 (17)	0.073 (2)	0.078 (2)	−0.0055 (15)	−0.0058 (15)	−0.0135 (17)
C19	0.0481 (15)	0.0690 (18)	0.0612 (17)	−0.0013 (13)	0.0076 (13)	−0.0123 (15)
C20	0.122 (3)	0.059 (2)	0.114 (3)	0.022 (2)	0.018 (2)	0.010 (2)
N1	0.0370 (12)	0.0694 (15)	0.0555 (13)	0.0035 (10)	0.0089 (10)	−0.0248 (11)
N2	0.0361 (11)	0.0558 (13)	0.0544 (13)	0.0033 (9)	0.0071 (9)	−0.0145 (10)
O1	0.0688 (12)	0.0634 (12)	0.0642 (12)	0.0126 (10)	0.0262 (10)	0.0144 (10)
O2	0.0382 (9)	0.0772 (13)	0.0585 (11)	0.0056 (9)	0.0038 (8)	−0.0159 (10)
S1	0.0428 (3)	0.0552 (4)	0.0435 (4)	0.0046 (3)	0.0095 (3)	−0.0070 (3)
Cl1	0.0827 (7)	0.1092 (8)	0.0920 (7)	0.0402 (6)	0.0573 (6)	0.0216 (6)
Cl1'	0.044 (3)	0.065 (4)	0.067 (4)	−0.011 (3)	0.001 (2)	−0.003 (3)

### Geometric parameters (Å, º)

**Table:**

C1—C2	1.375 (4)	C12—C13	1.364 (4)
C1—C6	1.378 (3)	C12—H12	0.9300
C1—H1	0.9300	C13—H13	0.9300
C2—C3	1.374 (4)	C14—C15	1.382 (3)
C2—H2	0.9300	C14—C19	1.385 (3)
C3—C4	1.373 (4)	C15—C16	1.374 (4)
C3—C20	1.510 (4)	C15—H15	0.9300
C4—C5	1.376 (4)	C16—C17	1.366 (4)
C4—H4	0.9300	C16—H16	0.9300
C5—C6	1.377 (3)	C17—C18	1.363 (4)
C5—H5	0.9300	C17—Cl1'	1.570 (5)
C6—S1	1.750 (2)	C17—H17	0.905 (19)
C7—N2	1.281 (3)	C18—C19	1.378 (4)
C7—C14	1.476 (3)	C18—H18	0.9300
C7—C8	1.484 (3)	C19—H19	0.9300
C8—C13	1.382 (3)	C20—H20A	0.9600
C8—C9	1.383 (3)	C20—H20B	0.9600
C9—C10	1.382 (3)	C20—H20C	0.9600
C9—H9	0.9300	N1—N2	1.385 (3)
C10—C11	1.374 (4)	N1—S1	1.639 (2)
C10—H10	0.9300	N1—H1A	0.877 (17)
C11—C12	1.365 (4)	O1—S1	1.4150 (18)
C11—Cl1	1.714 (3)	O2—S1	1.4296 (17)
			
C2—C1—C6	119.4 (3)	C8—C13—H13	119.6
C2—C1—H1	120.3	C15—C14—C19	118.3 (2)
C6—C1—H1	120.3	C15—C14—C7	120.5 (2)
C3—C2—C1	121.4 (3)	C19—C14—C7	121.2 (2)
C3—C2—H2	119.3	C16—C15—C14	120.9 (3)
C1—C2—H2	119.3	C16—C15—H15	119.5
C4—C3—C2	118.1 (3)	C14—C15—H15	119.5
C4—C3—C20	120.9 (3)	C17—C16—C15	119.5 (3)
C2—C3—C20	120.9 (3)	C17—C16—H16	120.3
C3—C4—C5	121.8 (3)	C15—C16—H16	120.3
C3—C4—H4	119.1	C18—C17—C16	121.1 (3)
C5—C4—H4	119.1	C18—C17—Cl1'	115.9 (3)
C4—C5—C6	119.0 (2)	C16—C17—Cl1'	123.1 (3)
C4—C5—H5	120.5	C18—C17—H17	118 (3)
C6—C5—H5	120.5	C16—C17—H17	121 (2)
C5—C6—C1	120.2 (2)	C17—C18—C19	119.5 (3)
C5—C6—S1	119.72 (19)	C17—C18—H18	120.3
C1—C6—S1	120.04 (19)	C19—C18—H18	120.3
N2—C7—C14	116.3 (2)	C18—C19—C14	120.8 (3)
N2—C7—C8	122.9 (2)	C18—C19—H19	119.6
C14—C7—C8	120.7 (2)	C14—C19—H19	119.6
C13—C8—C9	118.9 (2)	C3—C20—H20A	109.5
C13—C8—C7	119.1 (2)	C3—C20—H20B	109.5
C9—C8—C7	122.0 (2)	H20A—C20—H20B	109.5
C10—C9—C8	120.6 (2)	C3—C20—H20C	109.5
C10—C9—H9	119.7	H20A—C20—H20C	109.5
C8—C9—H9	119.7	H20B—C20—H20C	109.5
C11—C10—C9	118.4 (3)	N2—N1—S1	113.42 (16)
C11—C10—H10	120.8	N2—N1—H1A	121.1 (18)
C9—C10—H10	120.8	S1—N1—H1A	115.2 (17)
C12—C11—C10	121.8 (2)	C7—N2—N1	117.89 (19)
C12—C11—Cl1	118.3 (2)	O1—S1—O2	119.43 (12)
C10—C11—Cl1	119.9 (2)	O1—S1—N1	112.27 (12)
C13—C12—C11	119.3 (3)	O2—S1—N1	103.31 (11)
C13—C12—H12	120.4	O1—S1—C6	107.96 (11)
C11—C12—H12	120.4	O2—S1—C6	110.15 (12)
C12—C13—C8	120.9 (3)	N1—S1—C6	102.39 (11)
C12—C13—H13	119.6		
			
C6—C1—C2—C3	−0.4 (4)	C8—C7—C14—C15	−176.8 (2)
C1—C2—C3—C4	0.9 (4)	N2—C7—C14—C19	−175.2 (2)
C1—C2—C3—C20	−178.5 (3)	C8—C7—C14—C19	3.6 (4)
C2—C3—C4—C5	−0.5 (4)	C19—C14—C15—C16	0.1 (4)
C20—C3—C4—C5	179.0 (3)	C7—C14—C15—C16	−179.6 (2)
C3—C4—C5—C6	−0.5 (4)	C14—C15—C16—C17	−0.5 (4)
C4—C5—C6—C1	1.1 (4)	C15—C16—C17—C18	0.4 (5)
C4—C5—C6—S1	−178.9 (2)	C15—C16—C17—Cl1'	179.3 (3)
C2—C1—C6—C5	−0.7 (4)	C16—C17—C18—C19	0.2 (5)
C2—C1—C6—S1	179.3 (2)	Cl1'—C17—C18—C19	−178.8 (3)
N2—C7—C8—C13	64.3 (3)	C17—C18—C19—C14	−0.6 (5)
C14—C7—C8—C13	−114.3 (3)	C15—C14—C19—C18	0.5 (4)
N2—C7—C8—C9	−115.0 (3)	C7—C14—C19—C18	−179.8 (3)
C14—C7—C8—C9	66.4 (3)	C14—C7—N2—N1	178.9 (2)
C13—C8—C9—C10	−0.8 (4)	C8—C7—N2—N1	0.2 (4)
C7—C8—C9—C10	178.5 (2)	S1—N1—N2—C7	168.96 (19)
C8—C9—C10—C11	0.1 (4)	N2—N1—S1—O1	−50.0 (2)
C9—C10—C11—C12	0.4 (4)	N2—N1—S1—O2	−179.99 (18)
C9—C10—C11—Cl1	179.4 (2)	N2—N1—S1—C6	65.5 (2)
C10—C11—C12—C13	−0.1 (4)	C5—C6—S1—O1	17.3 (2)
Cl1—C11—C12—C13	−179.2 (2)	C1—C6—S1—O1	−162.68 (19)
C11—C12—C13—C8	−0.6 (4)	C5—C6—S1—O2	149.31 (19)
C9—C8—C13—C12	1.0 (4)	C1—C6—S1—O2	−30.7 (2)
C7—C8—C13—C12	−178.3 (2)	C5—C6—S1—N1	−101.3 (2)
N2—C7—C14—C15	4.5 (3)	C1—C6—S1—N1	78.7 (2)

### Hydrogen-bond geometry (Å, º)

**Table:**

*D*—H···*A*	*D*—H	H···*A*	*D*···*A*	*D*—H···*A*
N1—H1*A*···O2^i^	0.88 (2)	2.19 (2)	3.024 (3)	160 (2)
C1—H1···Cl1^ii^	0.93	2.91	3.694 (3)	143
C10—H10···Cl1′^iii^	0.93	2.76	3.476 (7)	134
C16—H16···O1^iv^	0.93	2.54	3.339 (3)	145

Symmetry codes: (i) −*x*+1, −*y*+2, −*z*+1; (ii) −*x*+1, *y*−1/2, −*z*+3/2; (iii) −*x*+2, *y*+1/2, −*z*+3/2; (iv) −*x*+2, −*y*+2, −*z*+1.
