# Supplementary figures and images for: Crystal structure of N′-[(E)-(4-chloro­phen­yl)(phen­yl)methyl­idene]-4-methyl­benzene­sulfono­hydrazide
Source: Acta Crystallogr E Crystallogr Commun. 2015 Jan 1;71(Pt 1):o45–6. doi: 10.1107/S2056989014026723 (PMC4331850; doi:10.1107/S2056989014026723)

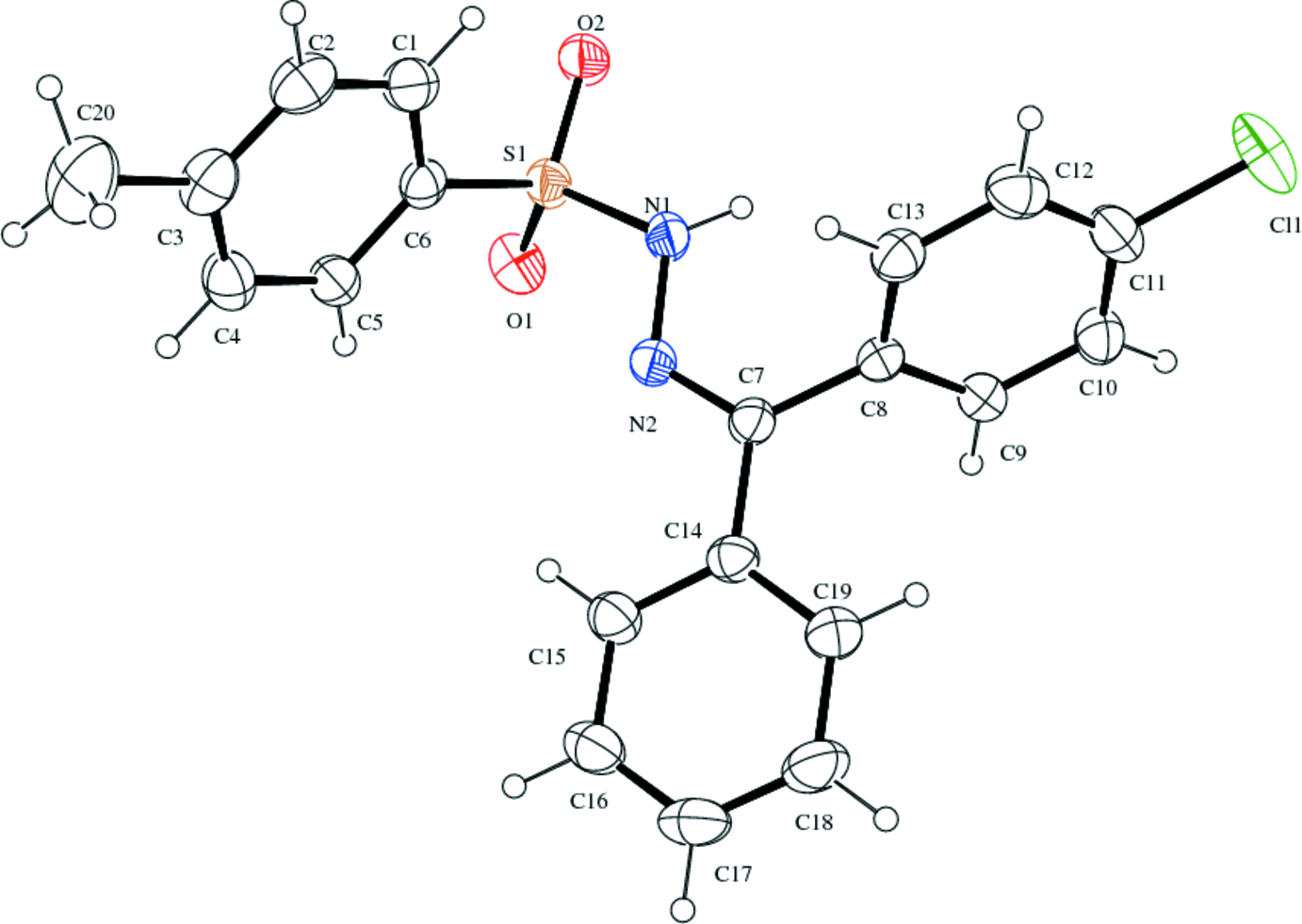

Supplement: Supplementary file 4 [file e-71-00o45-fig1.tif]

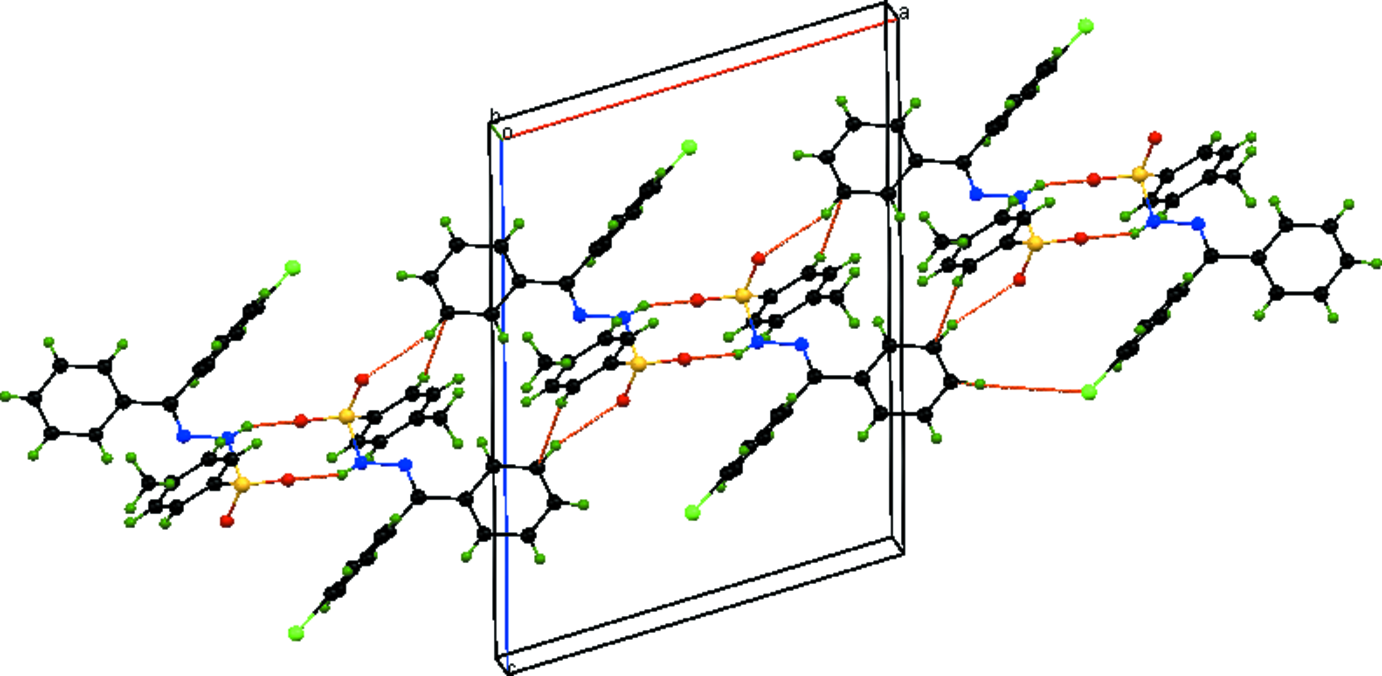

Supplement: Supplementary file 5 [file e-71-00o45-fig2.tif]
